# Persistence of virus-specific immune responses in the central nervous system of mice after West Nile virus infection

**DOI:** 10.1186/1471-2172-12-6

**Published:** 2011-01-20

**Authors:** Barbara S Stewart, Valerie L Demarest, Susan J Wong, Sharone Green, Kristen A Bernard

**Affiliations:** 1 Wadsworth Center, New York State Department of Health, Albany, NY, USA; 2 Department of Biomedical Sciences, School of Public Health, University at Albany, Albany, NY, USA; 3 Center for Infectious Disease and Vaccine Research, University of Massachusetts Medical School, Worcester, MA, USA; 4 Department of Pathobiological Sciences, School of Veterinary Medicine, University of Wisconsin-Madison, Madison, WI, USA

## Abstract

**Background:**

West Nile virus (WNV) persists in humans and several animal models. We previously demonstrated that WNV persists in the central nervous system (CNS) of mice for up to 6 months post-inoculation. We hypothesized that the CNS immune response is ineffective in clearing the virus.

**Results:**

Immunocompetent, adult mice were inoculated subcutaneously with WNV, and the CNS immune response was examined at 1, 2, 4, 8, 12 and 16 weeks post-inoculation (wpi). Characterization of lymphocyte phenotypes in the CNS revealed elevation of CD19^+ ^B cells for 4 wpi, CD138 plasma cells at 12 wpi, and CD4^+ ^and CD8^+ ^T cells for at least 12 wpi. T cells recruited to the brain were activated, and regulatory T cells (Tregs) were present for at least 12 wpi. WNV-specific antibody secreting cells were detected in the brain from 2 to 16 wpi, and virus-specific CD8^+ ^T cells directed against an immunodominant WNV epitope were detected in the brain from 1 to 16 wpi. Furthermore, these WNV-specific immune responses occurred in mice with and without acute clinical disease.

**Conclusions:**

Virus-specific immune cells persist in the CNS of mice after WNV infection for up to 16 wpi.

## Background

West Nile virus (WNV), a member of the family *Flaviviridae*, is a positive-sense, single-stranded RNA virus, which is maintained in a mosquito-bird enzootic cycle. Upon incidental infection with WNV, approximately 20% of humans experience a self-limiting illness called "West Nile fever", and less than 1% develop West Nile neuroinvasive disease (WNND) [1]. WNND is characterized by encephalitis, myelitis, and/or meningitis and can lead to death [2-4]. In addition to acute disease, long term sequelae occur in individuals recovering from West Nile fever and WNND [3,5-8]. The underlying mechanisms resulting in these sequelae remain unclear, but may partly be due to viral persistence.

Several studies provide evidence for persistence of WNV in humans. WNV RNA persists in urine of convalescent patients for as long as 6.7 years after disease onset [9]. In blood donors, WNV RNA is detected in blood as long as 104 days after index donation [10]. WNV-specific immunoglobulin M (IgM) persists in serum of patients with West Nile disease and WNV-positive blood donors for as long as 11 to 16 months [10-13]. In addition, IgM persists in cerebrospinal fluid of patients with WNND for as long as 5 months [14]. The long term persistence of IgM suggests that virus and/or viral antigen persists in the periphery and possibly in the CNS of immunocompetent humans infected with WNV.

The goal of the current study was to further our understanding of WNV persistence, using a mouse model in which WNV RNA persists in the CNS for up to 6 months post-inoculation [15]. We characterized the lymphocyte populations present in the CNS at various times post-inoculation. CD138+ plasma cells and CD4+ and CD8+ T cells were elevated in the CNS of mice for at least 3 months after infection with WNV. In addition, WNV-specific plasma cells and WNV-epitope specific CD8^+ ^T cells were present for up to 16 wpi, suggesting that WNV is able to persist in the CNS despite the presence of virus specific immune cells.

## Results

We previously showed that WNV RNA persists in the CNS of C57BL/6 (B6) mice for up to 6 months post-inoculation, and this persistence occurs in the face of active inflammation in the brain and a strong serum antibody response and in mice with subclinical infection [15]. Our goal in this study was to characterize this inflammation in the CNS during viral persistence in our B6 mouse model and to determine if the immune cells were virus-specific. Brains and spinal cords were harvested from mice, and the phenotypes of infiltrating CNS leukocytes were determined at 1, 2, 4, 8, 12 and 16 wpi. Since not all B6 mice exhibit West Nile disease [16], we distributed mice that had been sick during acute infection (7 to 14 days post-inoculation) evenly throughout each time point within an individual study (noted as open symbols in figures) in order not to bias the results. Although the numbers of sick mice were small, we did not observe any consistent correlation between disease and any cellular parameter.

**Figure 1:**
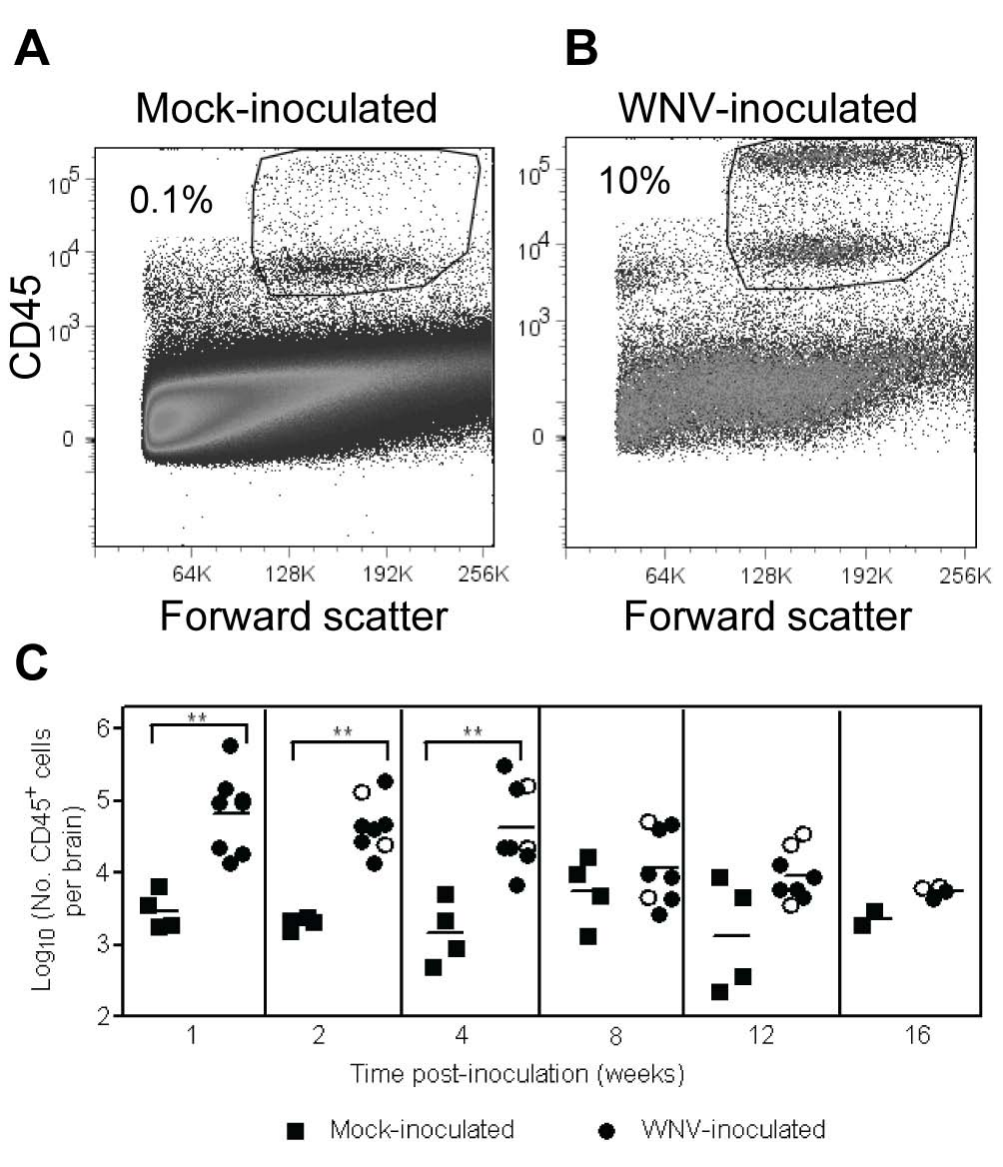
**WNV infection induces leukocyte infiltration in the CNS**. Adult, female B6 mice were inoculated SC with diluent (mock) or 10^3 ^PFU of WNV in the left rear footpad. At various times post-inoculation, two mock-inoculated and four WNV-inoculated mice were sacrificed and perfused with perfusion buffer. CNS mononuclear cells were collected, and flow cytometry was performed for various cell markers. Representative scatter plots and gating for flow cytometry are shown for leukocytes isolated from brains of (**A**) a mock-inoculated mouse and (**B**) a WNV-inoculated mouse. (**C**) Numbers of CD45^+ ^cells are reported per brain. Each data point represents an individual mouse, and solid horizontal lines represent the geometric means. Open symbols indicate mice that showed clinical signs of disease during acute illness. Two independent studies were performed for 1, 2, 4, 8 and 12 wpi, and these data were analyzed by Mann-Whitney *U *tests with *P *values indicated by: 0.001 < ** ≤0.01.

For all flow cytometric analyses, cells were gated on the entire population of CD45^+ ^cells, a pan-leukocyte marker. This population included a CD45^low ^population, which are quiescent resident microglial cells, and a CD45^high ^population, which are activated resident microglial cells and infiltrating leukocytes (Figure 1A and 1B). This gating ensured that differences between WNV-inoculated mice and mock-inoculated mice were not solely due to activated microglial cells, but due to infiltrating leukocytes and/or expansion of microglial cells.

### Phenotype of lymphocytes in the CNS

Elevated numbers of CD45^+ ^cells were observed in the brains of WNV-inoculated mice compared to mock-inoculated mice (Figure 1C). WNV-inoculated mice had 20- to 30-fold more CD45^+ ^cells in the CNS than mock-inoculated mice at 1, 2, and 4 wpi (*P *= 0.002). Similar levels of CD45^+ ^cells were observed in the spinal cords of WNV-inoculated mice (data not shown). After 4 wpi, the number of CD45^+ ^cells in the brains decreased with 2- to 7-fold more CD45^+ ^cells in the brains of WNV-inoculated mice than in mock-inoculated mice, but these differences were not statistically different. In addition, there was more variability from mouse-to-mouse at the later time points, which was consistent with our previous results of variable WNV RNA persistence and histologic lesions after 4 wpi [15].

**Figure 2:**
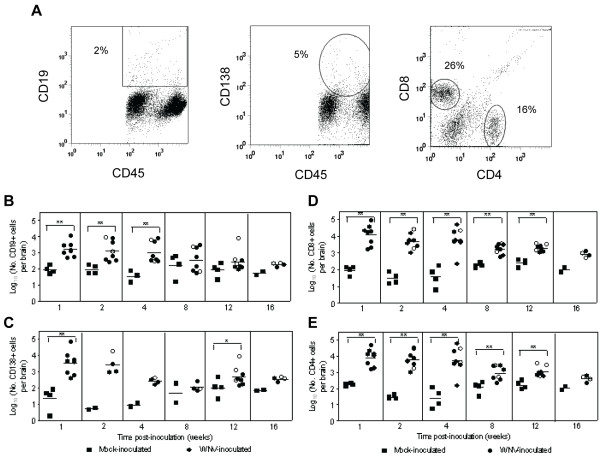
**B cells and T cells persist in the CNS of WNV-inoculated mice**. Adult, female B6 mice were inoculated SC with diluent (mock) or 10^3 ^PFU of WNV in the left rear footpad. At various times post-inoculation, two mock-inoculated and four WNV-inoculated mice were sacrificed and perfused with perfusion buffer. CNS mononuclear cells were collected, and flow cytometry was performed for various cell markers. Cells were gated on the CD45^+ ^population as shown in Figure 1. (**A**) Representative scatter plots are shown for CD19^+^, CD138^+^, CD4^+ ^and CD8^+ ^cells from WNV-inoculated mice. The numbers of (**B**) CD19^+^, (**C**) CD138^+^, (**D**) CD8^+^, and (**E**) CD4^+ ^cells per brain are shown at various times post-inoculation. Each data point represents an individual mouse, and solid horizontal lines represent the geometric means. Open symbols indicate mice that showed clinical signs of disease during acute illness. Two independent studies were performed for 1, 2, 4, 8 and 12 wpi for B, D and E and for 1 and 12 wpi for C, and these data were analyzed by Mann-Whitney *U *tests with *P *values indicated by: 0.001 < ** ≤0.01 and 0.01 < * ≤0.05.

We previously observed lymphocytic, plasmacytic inflammation in the brains of mice persistently infected with WNV RNA for up to 4 months post-inoculation [15]; therefore, we analyzed the leukocytes from brains for lymphocyte markers (Figure 2A). WNV-inoculated mice had significantly higher numbers of CD19^+ ^B cells for 1 to 4 wpi (*P *= 0.002) and CD138^+ ^plasma cells at 12 wpi (*P *= 0.02) in brains compared to mock-inoculated mice (Figure 2B and 2C). CD19^+ ^B cells declined over time after 4 wpi (Figure 2B). The highest numbers of CD138^+ ^plasma cells were observed at 1 and 2 wpi with geometric means of approximately 10^3.5 ^cells per brain for WNV-inoculated mice, and plasma cells in the brain slowly declined through 16 wpi (Figure 2C). Similar levels of CD19^+ ^B cells and CD138^+ ^plasma cells were observed in the spinal cords of WNV-inoculated mice (data not shown).

WNV-inoculated mice had significantly higher numbers of both CD8^+ ^and CD4^+ ^T cells in their brains than mock-inoculated mice from 1 through 12 wpi (*P *= 0.002-0.004; Figure 2D and 2E). Both CD8^+ ^and CD4^+ ^T cells remained constant in the brain through week 4 with geometric means of approximately 10^4 ^cells per brain for WNV-inoculated mice compared to approximately 10^2 ^cells per brain in mock-inoculated mice. After 4 wpi, numbers of T cells in the brain declined to approximately 10^3 ^cells per brain for WNV-inoculated mice. Similar results were found in the spinal cord except that infiltration was delayed by one week for CD4^+ ^T cells (peaking at 2 wpi) compared to infiltration in the brain (data not shown).

### Activated T cells and Tregs in the CNS

**Figure 3:**
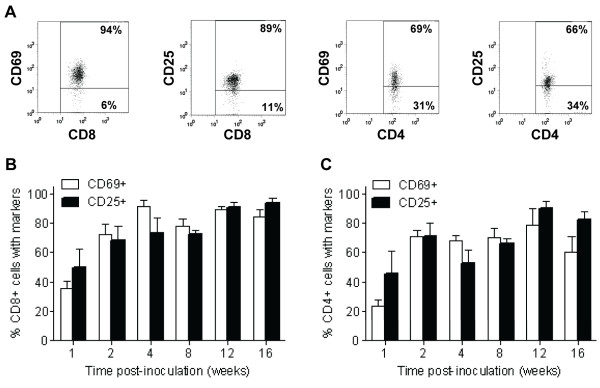
**T cells are activated in the CNS of WNV-inoculated mice**. Adult, female B6 mice were inoculated SC with 10^3 ^PFU of WNV in the left rear footpad. At various times post-inoculation, four WNV-inoculated mice were sacrificed and perfused with perfusion buffer. CNS mononuclear cells were collected, and flow cytometry was performed for various cell markers. Cells were gated on the CD45^+ ^population as shown in Figure 1. (**A**) Representative scatter plots are shown for activation markers, CD69 or CD25, on cells gated for the CD4^+ ^or CD8^+ ^population from WNV-inoculated mice. The percentage of (**B**) CD8^+ ^T cells and (**C**) CD4^+ ^T cells expressing either CD69 or CD25 in the brains of WNV-inoculated mice are shown at various times post-inoculation. Bars and error bars represent the average and SD of four mice.

Elevated numbers of T cells were present in the CNS, but we previously showed that clearance of WNV RNA was delayed until after 2 months post-inoculation [15]. Thus, we questioned if the T cells in the CNS were activated, and if Tregs were present. We examined CD8^+ ^and CD4^+ ^T cells in the CNS for two activation markers, CD69 and CD25 (Figure 3A). Activated CD8^+ ^and CD4^+ ^T cells were observed by 1 wpi with approximately 50% of these cells expressing at least one activation marker (Figure 3B and 3C). The percentage of activated CD8^+ ^T cells peaked at approximately 90% at 4 wpi and remained fairly constant through 16 wpi (Figure 3B). The percentage of activated CD4^+ ^T cells reached 70% at 2 wpi and 90% at 12 wpi (Figure 3C). In summary, CD8^+ ^and CD4^+ ^T cells in the brain were activated for at least 16 wpi.

**Figure 4:**
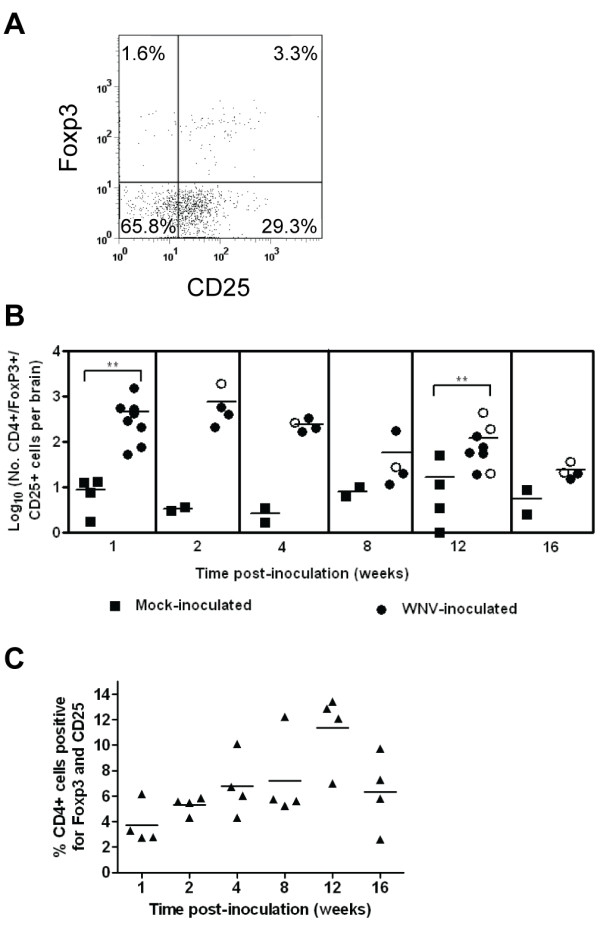
**Tregs are present in the CNS of WNV-inoculated mice**. Adult, female B6 mice were inoculated SC with diluent (mock) or 10^3 ^PFU of WNV in the left rear footpad. At various times post-inoculation, two mock-inoculated and four WNV-inoculated mice were sacrificed and perfused with perfusion buffer. CNS mononuclear cells were collected, and flow cytometry was performed for various cell markers. Cells were gated on the CD45^+ ^population as shown in Figure 1. (**A**) Representative scatter plot is shown for Treg markers, Foxp3 and CD25, on cells gated for the CD4^+ ^population from a WNV-inoculated mouse. (**B**) The numbers of CD4^+^/Foxp3^+^/CD25^+ ^cells per brain are shown at various times post-inoculation. Each data point represents an individual mouse, and solid horizontal lines represent the geometric means. Open symbols indicate mice that showed clinical signs of disease during acute illness. The data point on the x-axis is negative. Two independent studies were performed for 1 and 12 wpi, and these data were analyzed by Mann-Whitney *U *tests with *P *values indicated by: 0.001 < ** ≤0.01. (**C**) The percentage of CD4^+ ^cells expressing Foxp3 and CD25 in the brains of WNV-inoculated mice is shown at various times post-inoculation. Each data point represents an individual mouse, and solid horizontal lines represent the means. The groups from each time point were significantly different by a Kruskal-Wallis test (*P *= 0.05), and the group at 12 wpi was significantly different from the groups at 1, 2 and 4 wpi by Mann-Whitney *U *tests (*P *< 0.05).

We hypothesized that Tregs may delay viral clearance by inhibiting the CD8^+ ^T cell response; therefore, we examined Tregs, defined as cells expressing CD4, CD25, and Foxp3 [17], in the brains of mice over time (Figure 4A). We observed Tregs as early as 1 wpi in the brains of WNV-inoculated mice (50-fold greater than in mock-inoculated mice; *P *= 0.002) and persisting for at least 12 wpi (8-fold greater than in mock-inoculated mice; *P *= 0.008) (Figure 4B). The percentages of CD4^+ ^T cells exhibiting the CD25^+^Foxp3^+ ^phenotype increased 3-fold from 1 to 12 wpi (*P *= 0.03) (Figure 4C). The highest percentage of Tregs at 12 wpi (mean of 11.4%) correlated with the highest percentage of activated CD4^+ ^T cells (Figure 3C) and relatively high levels of CD4^+ ^T cells in brains of WNV-inoculated mice (Figure 2E).

### WNV-specific immune cells in the CNS

In our phenotypic analysis, we observed CD138^+ ^plasma cells, and activated CD8^+ ^and CD4^+ ^T cells in the CNS of WNV-inoculated animals for at least 12 wpi. However, despite an active immune response in the CNS, our previous results showed that WNV RNA continues to persist in the CNS of approximately half of the mice at 3 months post-inoculation and a quarter of the mice at 4 months post-inoculation [15].

**Figure 5:**
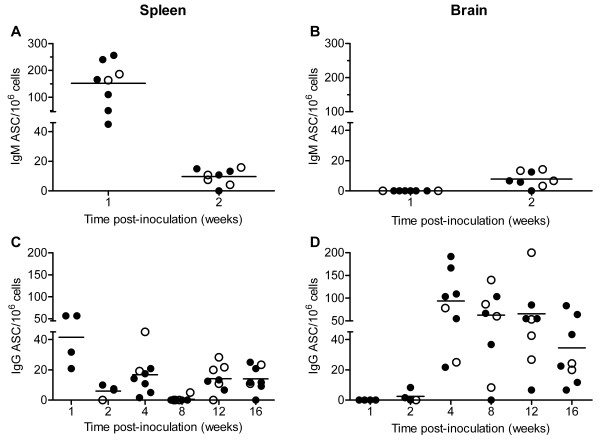
**WNV-specific ASC persist in brains for at least 16 wpi in WNV-inoculated mice**. Adult, female B6 mice were inoculated SC with diluent (mock) or 10^3 ^PFU of WNV in the left rear footpad. At various times post-inoculation, two mock-inoculated and four WNV-inoculated mice were sacrificed and perfused with perfusion buffer. Brain and splenic mononuclear cells were collected, and ELISPOT assays for ASC were performed using WNV and irrelevant antigens. The numbers of IgM ASC in the (**A**) spleen and (**B**) brain and the numbers of IgG ASC in the (**C**) spleen and (**D**) brain are reported. Each data point represents a single mouse, and solid horizontal lines represent the means. Open symbols indicate mice that displayed signs of clinical disease during the acute illness. Data points on the x-axis are negative. Data are from one to two independent studies. Results from mock-inoculated mice were negative and are not shown.

In order to determine whether the inflammatory response that we detected was WNV-specific, we examined the humoral response by performing ELISPOT assays for WNV-specific IgM and IgG antibody secreting cells (ASC) in brains and spleens. At 1 wpi, IgM ASC specific for WNV were detected at relatively high numbers in the spleen (average = 150 IgM ASC/10^6 ^cells), but none were detected in the brain (Figure 5A and 5B). By 2 wpi, seven of eight WNV-inoculated mice had IgM ASC in brains and spleens (average = 10 IgM ASC/10^6 ^cells). By 4 wpi, no IgM ASC were detected in the brains or spleens (data not shown), which was most likely due to class switching to IgG. WNV-specific IgG ASC were first detected in spleens at 1 wpi and in brains at very low levels at 2 wpi (Figure 5C and 5D). By 4 wpi, there were 5-fold more IgG ASC in brains (average of 100 IgG ASC/10^6 ^cells) than in spleens (average of 20 IgG ASC/10^6 ^cells). The numbers of WNV-specific IgG ASC remained relatively constant in the two tissues through 16 wpi except at 8 wpi when only one of eight mice had detectable IgG ASC in the spleen. Seven of eight mice were positive for IgG ASC in the spleens at 12 and 16 wpi, suggesting that the immune response was stimulated between 8 and 12 wpi.

**Figure 6:**
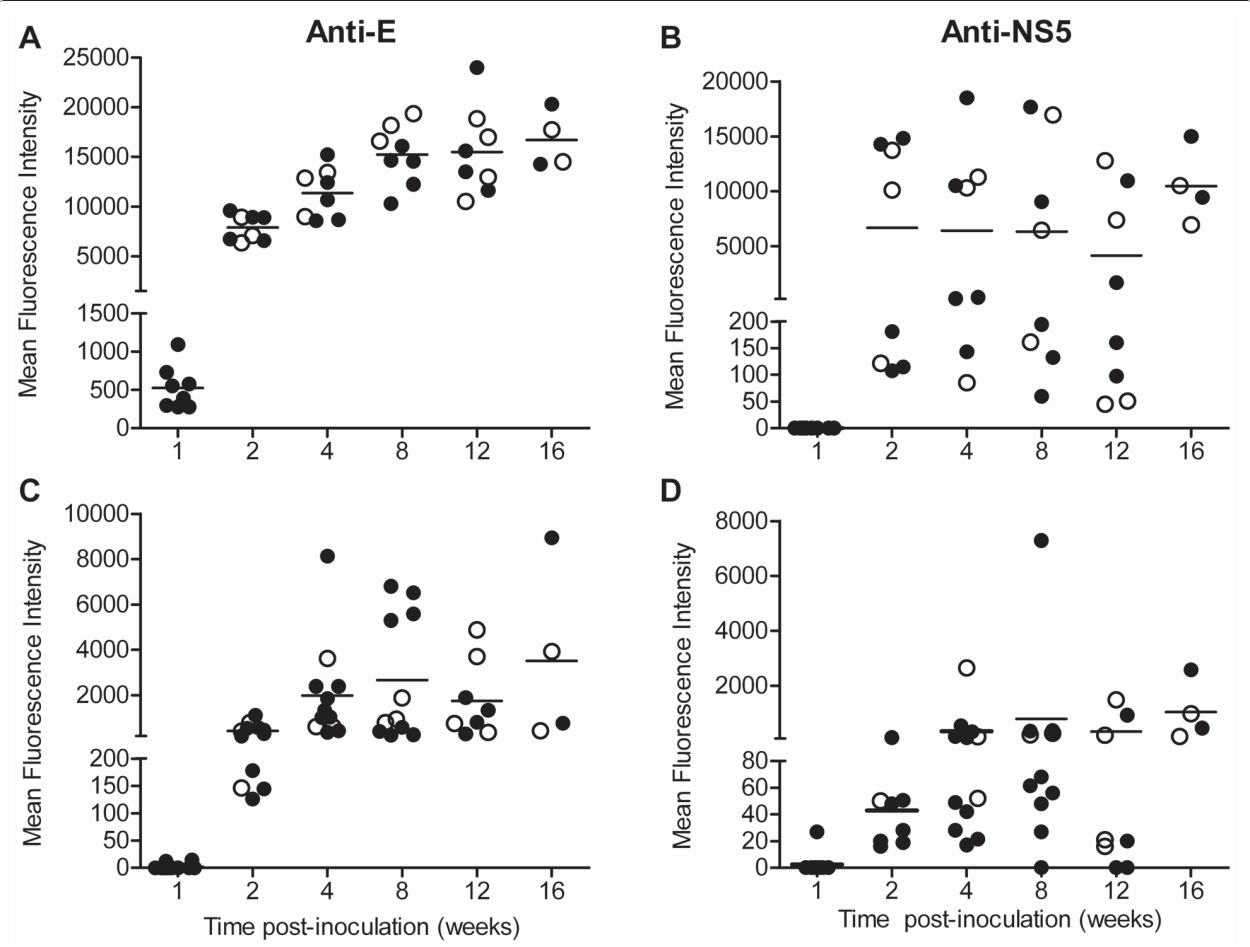
**WNV-specific antibodies are present in brains for at least 16 wpi in WNV-inoculated mice**. Adult, female B6 mice were inoculated SC with diluent (mock) or 10^3 ^PFU of WNV in the left rear footpad. At various time points post-inoculation, two mock-inoculated and four WNV-inoculated mice were sacrificed and perfused with perfusion buffer. (**A**and **B**) Sera and (**C**and **D**) supernatant from homogenized brain were tested for antibodies against (**A**and **C**) WNV E and (**B**and **D**) WNV NS5 proteins using MIA. Each data point represents an individual mouse, and solid horizontal lines represent the means. Open symbols indicate mice that displayed signs of clinical disease during the acute illness. Data points on the x-axis are negative. Data are from one to two independent studies. Results from mock-inoculated mice were negative and are not shown.

We confirmed the presence of intrathecal antibody in the CNS by measuring antibody specific for WNV E and NS5 proteins in supernatants from CNS homogenates. The kinetics of WNV-specific antibodies in the brain were very similar to the kinetics of the antibody response measured in the serum; however, the levels of antibody were approximately 50-fold lower in brains than in sera (Figure 6). Antibodies to E and NS5 were first observed in brains at 1 wpi in 25% and 12% of mice, respectively (Figure 6C and 6D). At this time point, ASC were not observed in brains (Figure 5B and 5D), which is most likely due to differences in assay sensitivity. Antibodies to E and NS5 were also observed in spinal cords of mice, and the kinetics and levels of antibodies were very similar to the results in the brains (data not shown).

**Figure 7:**
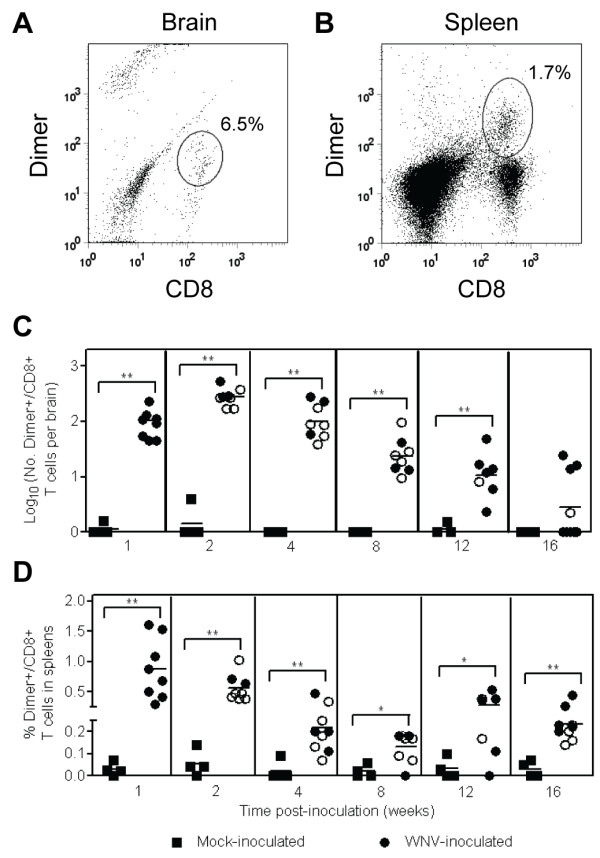
**WNV-specific CD8^+ ^T cells persist in brains for up to 16 wpi in WNV-inoculated mice**. Adult, female B6 mice were inoculated SC with diluent (mock) or 10^3 ^PFU of WNV in the left rear footpad. At various times post-inoculation, two mock-inoculated and four WNV-inoculated mice were sacrificed and perfused with perfusion buffer. CNS and splenic mononuclear cells were collected, and flow cytometry was performed for CD45, CD8 and MHC class I dimer for a WNV dominant epitope (SSVWNATTA) and an irrelevant epitope. Cells were gated on the CD45^+ ^population as shown in Figure 1. Representative scatter plots are shown for CD8 and WNV-specific dimer in the (**A**) brain and (**B**) spleen from WNV-inoculated mice. The numbers of CD8^+ ^T cells in the (**C**) brain and the percentages of CD8^+ ^T cells in the (**D**) spleen that were specific for the WNV epitope are reported. Values for specific CD8^+ ^T cells were calculated by subtracting background values for the irrelevant peptide from values for the WNV peptide. Each data point represents an individual mouse, and solid horizontal lines represent the geometric means in (**C**) and means in (**D**). Open symbols indicate mice that displayed signs of disease during acute illness. Data points on the x-axis are negative. Two independent studies were performed, and these data were analyzed by Mann-Whitney *U *tests with *P *values indicated by: 0.001 < ** ≤0.01 and 0.01 < * ≤0.05.

We next examined the WNV-specific CD8^+ ^T cell response to an immunodominant WNV epitope, SSVWNATTA in NS4B [18,19], using MHC class I dimer staining in brains and spleens (Figure 7A and 7B). WNV epitope-specific CD8^+ ^T cells were significantly higher in the brains of WNV-inoculated mice compared to mock-inoculated mice from 1 to 12 wpi (*P *= 0.004-0.005; Figure 7C). WNV epitope-specific CD8^+ ^T cells peaked at 2 wpi (average of 300 cells per brain) (Figure 7C), representing approximately 5% of all CD8^+ ^T cells in the brain (data not shown). By 16 wpi in the brain, WNV epitope-specific CD8^+ ^T cells were detected in four of eight mice. In the spleen, WNV epitope-specific CD8^+ ^T cells were significantly higher in WNV-inoculated mice compared to mock-inoculated mice from 1 to 16 wpi (*P *= 0.004-0.02; Figure 7D). In contrast to the brain, epitope-specific CD8^+ ^T cells in the spleen were highest at 1 wpi and declined through 8 wpi (Figure 7D). At 12 and 16 wpi, there was an increase in WNV epitope-specific CD8^+ ^T cells in the spleen, suggesting that the immune response was stimulated between 8 and 12 wpi as was observed in the independent study that measured ASC in the spleen (Figure 5C).

## Discussion and Conclusions

Our studies are the first to demonstrate the retention of WNV-specific immune cells in the CNS in an immunocompetent mouse model. Immune cells were recruited and maintained in the CNS for at least 12 wpi, including plasma cells, activated T cells and Tregs. Additionally, WNV-specific ASC and CD8^+ ^T cells specific for a dominant epitope of WNV were detected in the brains of mice for up to16 wpi, including mice without clinical disease. The presence of specific immune cells is consistent with our previous studies, which showed that WNV persists in the CNS in a subset of animals as infectious virus for 4 months post-inoculation and viral RNA for 6 months post-inoculation [15]. Overall these results suggest that the presence of virus-specific immune cells in the CNS is not sufficient for viral clearance.

Our current study was limited in that we were unable to correlate WNV persistence and cellular phenotype in the CNS within the same individual mouse because we harvested the entire brain and spinal cord for cell analysis. On the other hand, we have used identical methods, including mouse strain, sex and age, viral dose, inoculation route, and WNV strain, as our previous study [15], which has been repeated with very similar results in a number of independent studies. Thus, although we do not have correlative data, we have strong evidence that WNV persists as infectious virus in all mice at 1 month post-inoculation and as RNA in approximately 25% of mice at 4 months post-inoculation.

We observed no obvious deficiencies in the immune cell infiltrate in the CNS to account for the observed viral persistence in B6 mice [15]. Previous studies have shown that T cells play a crucial role in clearing WNV from CNS tissues. Mice that lack CD8^+ ^T cells have higher viral burdens, and infectious virus can be recovered for several weeks following initial infection [20,21]. Additionally, CD4^+ ^T cells are important for sustaining and maintaining primary CD8^+ ^T cells responses in the brain [22]. B cells and antibody play a critical role during WNV infection. μMT mice, which are deficient in B cells, have higher viremia and viral loads in the CNS, suggesting that B cells directly limit the early replication of virus in the CNS [23]; however, CD8^+ ^T cells are responsible for viral clearance in the CNS tissues [20]. In our studies, elevated numbers of CD4^+ ^and CD8^+ ^T cells, and plasma cells were present in the CNS during the time course of the studies.

In addition, we demonstrated that T cells in the brains of mice had early (CD69) and late (CD25) activation markers through 16 wpi. Typically, CD69 is transiently upregulated during early T cell activation; however, in our studies, CD69 was chronically expressed on both CD4^+ ^and CD8^+ ^T cells in the brain, which suggests either an ongoing stimulation of resident T cells or fresh recruits infiltrating into the CNS. The continual expression of CD69 on T cells occurs in other viral models, including dengue, neurotropic influenza and mouse hepatitis viruses, and is a common feature of CD8^+ ^T cells retained in the CNS [24-26]. It has been postulated that the chronic expression of CD69 identifies cells that are in a state of anergy or non-responsiveness due to prolonged antigen exposure [24,25,27,28]. In our studies, both CD69 and CD25 expressing T cells were recruited and maintained at high percentages in the CNS, suggesting that there is ongoing stimulation of these cells via virus and/or antigen. Alternatively, there may be bystander activation of non-specific cells and/or a failure to turn off the activation state.

We observed long term persistence of WNV-specific CD8^+ ^T cells in brains of mice, which supports the model of ongoing stimulation. At the end of the studies (16 wpi), half of the mice had detectable CD8^+ ^T cells specific for an immunodominant epitope in WNV in the brain. These findings are consistent with our previous finding that WNV RNA and infectious virus persists in the CNS at 16 wpi in 25% and 12% of mice, respectively [15]. Furthermore, WNV-specific ASC were first detected in brains at 2 wpi and persisted for up to 16 wpi.

The trafficking and persistence of ASC in the CNS also occurs in other viral models. We observed that antiviral IgM and IgG ASC in the spleen preceded the detection of ASC in the brain, suggesting that B cells from the periphery are activated and subsequently traffic to the CNS where they differentiate into ASC, as occurs for mouse hepatitis virus [29]. Similar to our results, virus-specific ASC are detected in mouse brains for one year after infection with Sindbis virus [30] and for 90 days after infection with mouse hepatitis virus [29,31]. Since we detected WNV RNA for up to six months in the CNS [15], the retention of WNV-specific ASC within the CNS suggests that these cells contribute to continual viral suppression via long term production of intrathecal antibody as observed with other encephalitic viruses [30-32].

The persistence of WNV in the CNS in the face of a specific local immune response raises the following question: why is viral clearance from the CNS delayed for 6 months in a subset of animals? We propose that the persistent WNV infection in the CNS is a low level, smoldering infection that is prevented from spreading by the presence of WNV-specific intrathecal antibody. Although T cells are activated, and virus-specific CD8^+ ^T cells are present, we speculate that they are inhibited in order to prevent excessive damage to neurons. This inhibition may be due to Tregs, which we observed in the CNS during the entire course of our studies. Lower levels of Tregs are associated with greater disease in humans and mice [33], and thus, Tregs may be important in inhibiting immunopathology in the CNS and allowing viral persistence. Future studies will focus on testing this model and furthering our understanding of the CNS immune response during persistent viral infections.

## Methods

### Virus and cells

WNV was produced from a full-length cDNA clone of a strain isolated in 2000 in New York as previously described [34]. Viral titers of stocks were determined by plaque assay on Vero cells (ATCC #CCL-81) as previously described [16]. Serum-free WNV stocks were harvested from Vero cells incubated in VP medium (Gibco^® ^Invitrogen, Carlsbad, CA).

### Mice

Five-week-old female B6 mice (Taconic, Germantown, NY) were acclimatized for at least one week in the BSL-3 facility. At six- to seven-weeks of age, mice were inoculated subcutaneously (SC) in the left rear footpad with diluent (mock) or 10^3 ^plaque forming units (PFU) of WNV as previously described [16]. After inoculation, all mice were observed for clinical disease daily for the entire study, and they were weighed daily for at least 2 wpi and one to three times per week for the remainder of the study. Clinical signs included ruffled fur, hunching, ataxia, and weakness. A mouse was considered to have clinical West Nile disease if at least one of the following criteria was met: 1) ≥10% weight loss; 2) clinical signs for at least two days. Mice that exhibited severe disease were euthanized. No clinical signs or weight loss were observed in mock-inoculated mice. All WNV-inoculated mice were seropositive for WNV at the time of sacrifice. At various times post-inoculation, mice were sacrificed and transcardially perfused with 60 ml phosphate buffered saline (PBS) plus 1% fetal bovine serum (FBS) [perfusion buffer], and tissues were harvested. All studies were approved by the Institutional Animal Care and Use Committee and followed criteria established by the National Institutes of Health.

### Isolation of mononuclear cells

Brains, spinal cords, and spleens were homogenized between glass slides with 10 ml of RPMI 1640 (RPMI, Gibco^® ^Invitrogen) plus 5% FBS. Spinal cords from four WNV-inoculated mice were pooled in order to obtain enough cells for analysis. Homogenates were pressed through a 100 micron cell strainer and centrifuged at 1200 × g for 5 minutes at 4°C. The cell pellet was resuspended in RPMI plus 5% FBS, placed over a 30/70% Percoll (Sigma-Aldrich, St. Louis, MO) step gradient, and centrifuged at 400 × g for 25 minutes at 4°C. The mononuclear cells were collected at the interface, washed two times, and resuspended in RPMI plus 5% FBS.

### Flow cytometry

For cell surface analysis, mononuclear cells (10^6 ^cells/test) were incubated with Fc block (BD Biosciences, San Jose, CA) and stained for surface antigen with two or more fluorochrome-labeled monoclonal antibodies directed against CD4, CD8, CD19, CD138, (BD Biosciences), CD25, CD45, Foxp3 (eBioscience, San Diego, CA), and CD69 (Caltag, Burlingame, CA). Cells that were stained for CD4 and CD25 were permeabilized and intracellularly stained using a Foxp3 staining buffer kit (eBioscience) according to manufacturer's protocol. Cells were fixed with 2% paraformaldehyde at 4°C overnight prior to analysis with FACSAria flow cytometer (BD Biosciences). For CNS samples, one million live events were counted. For splenocytes, 75,000 live events were counted. For analysis, all samples were first gated on CD45 as described previously for mouse brain samples [29]. Isotype matched irrelevant antibodies were used as controls. Data were analyzed using FlowJo software (v.7, Tree Star, Ashland, OR).

### ELISPOT assay for ASC

For WNV-specific ELISPOT assays for ASC, plates (96-well nitrocellulose; Millipore, Billerica, MA) were coated with serum-free WNV (10^6 ^PFU/well) or conditioned medium (irrelevant antigen) and then blocked with RPMI plus 10% FBS. Mononuclear cells were isolated from brains and spleens of mock- or WNV-inoculated mice as described above, added to wells (6.25 × 10^3 ^to 4 × 10^5 ^cells/well) in triplicate, and incubated overnight. ASC spots were developed by sequential addition of biotinylated anti-IgM or anti-IgG (Vector Laboratories, Burlingame, CA), horseradish peroxidase-conjugated strepavidin (Vector Laboratories), and 3-amino-9-ethyl carbazole substrate (BD Biosciences) and counted using a dissecting microscope. The numbers of specific ASC were determined by subtraction of the average number of spots in the irrelevant antigen wells (range: 0-2 in brains, 0-14 in spleens) from the average number of spots in the WNV antigen wells.

### Microsphere immunoassay (MIA) for WNV-specific antibody in tissues

Sera, brains and spinal cords were harvested from mock- or WNV-inoculated mice that were sacrificed and perfused at various times post-inoculation as described above. Tissues were processed as previously described [16]. Briefly, tissues were harvested and weighed, and RPMI was added to make a 20% homogenate. Sera and tissue supernatants were heat-inactivated for 1 hour at 56°C and tested at 1:100 and 1:10 dilutions, respectively, using fluorescent MIA for WNV envelope (E) and non-structural 5 (NS5) proteins as previously described [15,35]. Cutoff values for positive samples were calculated as the average mean fluorescence intensity of sera or tissue supernatants from mock-inoculated mice plus three standard deviations (Microsoft^®^Office Excel, Microsoft Corporation, Seattle, WA).

### CD8^+ ^T cell MHC class I dimer staining

Recombinant soluble dimeric mouse H-2D^b^:Ig (BD Biosciences) was incubated with one of the following peptides at 640 molar excess: the immunodominant WNV epitope (SSVWNATTA) [18,19] or an irrelevant H-2D^b ^restricted influenza virus epitope (NP-366-374, Anaspec, San Jose, CA). Anti-mouse IgG1 (BD Biosciences) and isotype control were added and incubated sequentially. Mononuclear cells from brains and spleens of mock- or WNV-inoculated mice were isolated as described above and added to the peptide-dimer mixture (10^6 ^cells/test). Cells were then surface stained with anti-CD8 and anti-CD45 and analyzed by flow cytometry as described above. Each tissue sample was tested with WNV peptide and the influenza virus peptide, and the WNV-specific CD8^+ ^cells were determined by subtraction of background staining (influenza virus peptide) from the specific staining (WNV peptide).

### Statistical analysis

Numbers of cells per organ were analyzed for data with 2 independent studies. A one-tailed Mann-Whitney *U *test was used to compare data from mock- and WNV-inoculated mice, and a two-tailed Mann-Whitney *U *test was used to compare data from two different time points for WNV-inoculated mice (GraphPad, San Diego, CA). A two-tailed Kruskal-Wallis test was used to compare more than two groups (GraphPad). For all analyses, a *P*-value of less than 0.05 was considered significant.

## Authors' contributions

BSS participated in study design and data interpretation, carried out all animal studies, performed immunologic assays, and drafted the manuscript. VLD performed immunologic assays. SJW participated in data analysis. SG participated in study design and revised the manuscript. KAB conceived of the study, participated in study design and data interpretation, supervised the animal studies, and revised the manuscript. All authors read and approved the final manuscript.
